# Acute myocardial infarction and transient elevated anticardiolipin antibody in a young adult with possible familial hypercholesterolemia: a case report: Anticardiolipin antibody and myocardial infarction

**DOI:** 10.1186/s12872-019-1135-y

**Published:** 2019-06-27

**Authors:** Xin Su, Aqian Wang, Hai Zhu, Hongling Su, Yichao Duan, Shanlian Wu, Min Zhang, Yan Huang, Xing Zhou, Yunshan Cao

**Affiliations:** 1 Department of Cardiology, Gansu Provincial Hospital, Lanzhou University, Lanzhou, 730000 People’s Republic of China; 2 School of Clinical Medicine, Gansu University of Chinese Medicine, Lanzhou, 730000 People’s Republic of China; 3 School of Clinical Medicine, Ningxia Medical University, Lanzhou, 730000 People’s Republic of China; 4 Department of Pathology, Gansu Provincial Hospital, Lanzhou University, Lanzhou, 730000 People’s Republic of China; 5 Department of Radiology, Gansu Provincial Hospital, Lanzhou University, Lanzhou, 730000 People’s Republic of China

**Keywords:** Familial hypercholesterolemia, Thrombosis, Myocardial infarction, Anticardiolipin antibody

## Abstract

**Background:**

Familial hypercholesterolemia (FH) can lead to premature coronary heart disease. Anticardiolipin antibody may be a contributor for thrombosis. Here, we report an adult with possible FH suffered from premature myocardial infarction that may be triggered by transient increased anticardiolipin antibody.

**Case presentation:**

A 29-year-old male had presented with a history of 2-h chest pain and numbness of left upper arm before 5 days. The electrocardiogram (ECG) had demonstrated inferior wall myocardial infarction (MI). Five days later he was admitted to our hospital and diagnosed as acute MI and possible FH (premature coronary heart disease, low density lipoprotein cholesterol of 5.90 mmol/L) with increased anticardiolipin antibody (up to 120 RU/ml). Other auto-antibodies including β2-glicoprotein antibodies IgM, IgA, IgG, lupus anticoagulant (LA), antinuclear antibodies, anti-myocardial antibody were normal. Coronary artery angiography (CAG) showed right coronary artery was total occlusion from the middle segment. Then he underwent percutaneous coronary intervention with a stent. Four days later, he was discharged with complete recovery. CAG showed intra-stent restenosis and anticardiolipin antibody level was normal and the patient had no any symptoms at 6-month follow-up.

**Conclusions:**

Transient elevated anticardiolipin antibody may be a trigger or biomarker of cardiac thrombotic events in younger atherosclerotic patients.

## Background

It is well known that the incidence of premature cardiovascular disease (CVD) is low. Previous studies have revealed that patients aged less than 40 years old only account for 1.2% of all patients with MI [1, 2]. Numerous studies have reported that multiple risk factors relate to ST segment elevated MI (STEMI) including male, smoking state, family history of CVD, dyslipidemia, hypertension, and diabetes mellitus (DM) in patients aged < 40 years [3–7]. Familial hypercholesterolemia (FH) as a type of dyslipidemia is one of the most common risk factors in patients with premature atherosclerotic cardiovascular disease (ASCVD) [8, 9]. In addition, previous evidence has proved that the presence of antiphospholipid antibodies (aPL) increases the thrombotic risk and the decreased titers or the disappearance of aPL closely relates to better prognosis [10–13]. Thus, a transient increase of anticardiolipin antibody induced by bacteria or viruses infection may contribute to the risk of thrombosis in patient with possible FH [14, 15].

## Case presentation

**Fig. 1:**
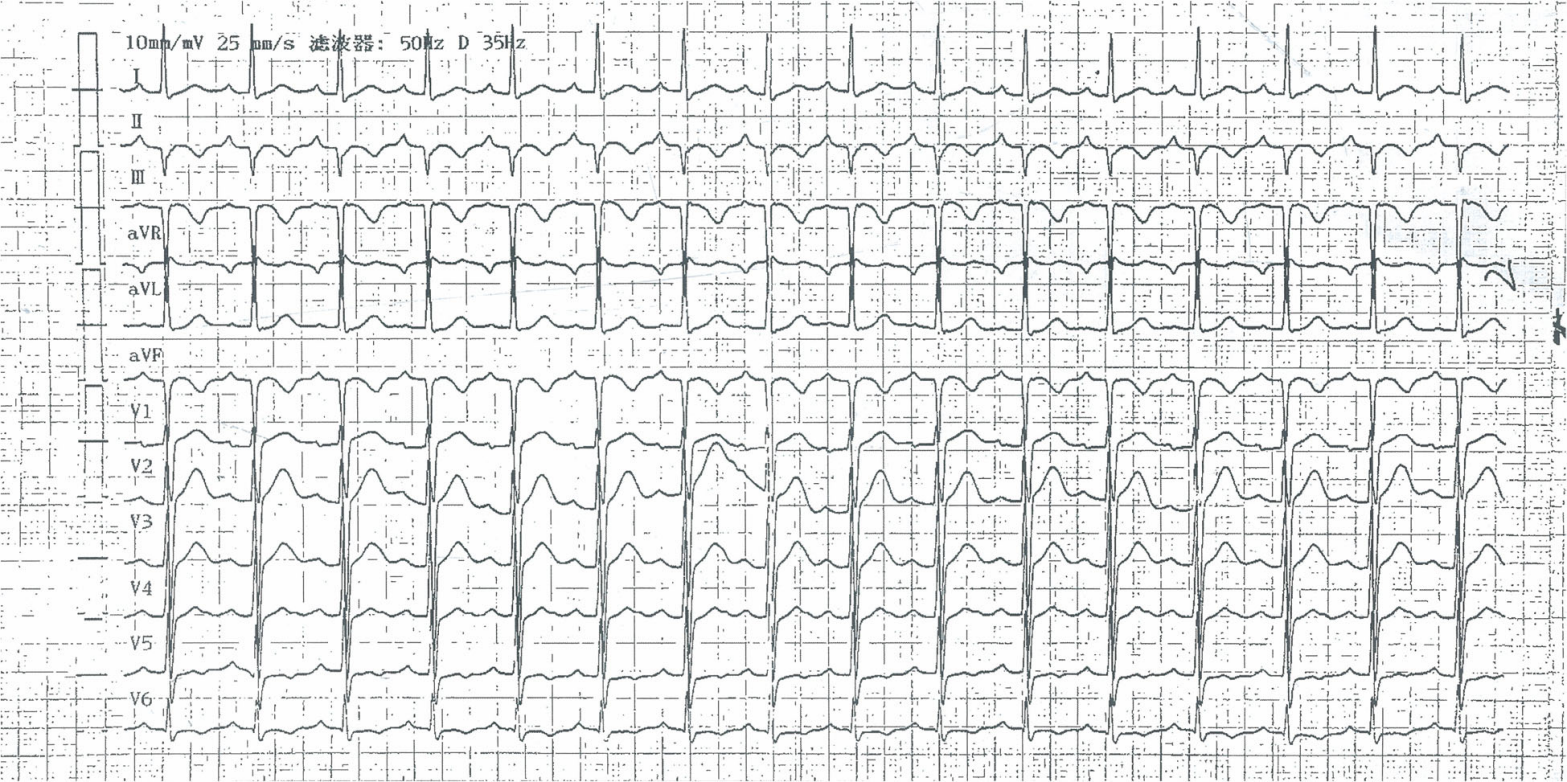
Electrocardiogram at admission

**Fig. 2:**
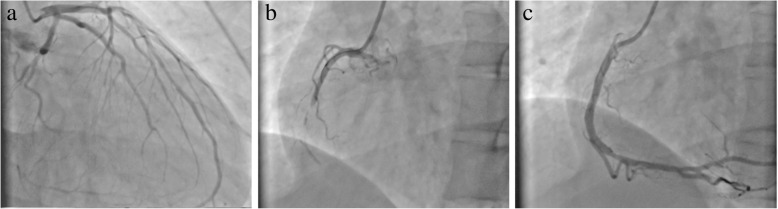
Coronary artery angiography and percutaneous coronary intervention (PCI). **a**. Left anterior descending artery (LAD); **b**. Right coronary artery (RCA); **c**. Implantation of stent in the middle right coronary artery

**Fig. 3:**
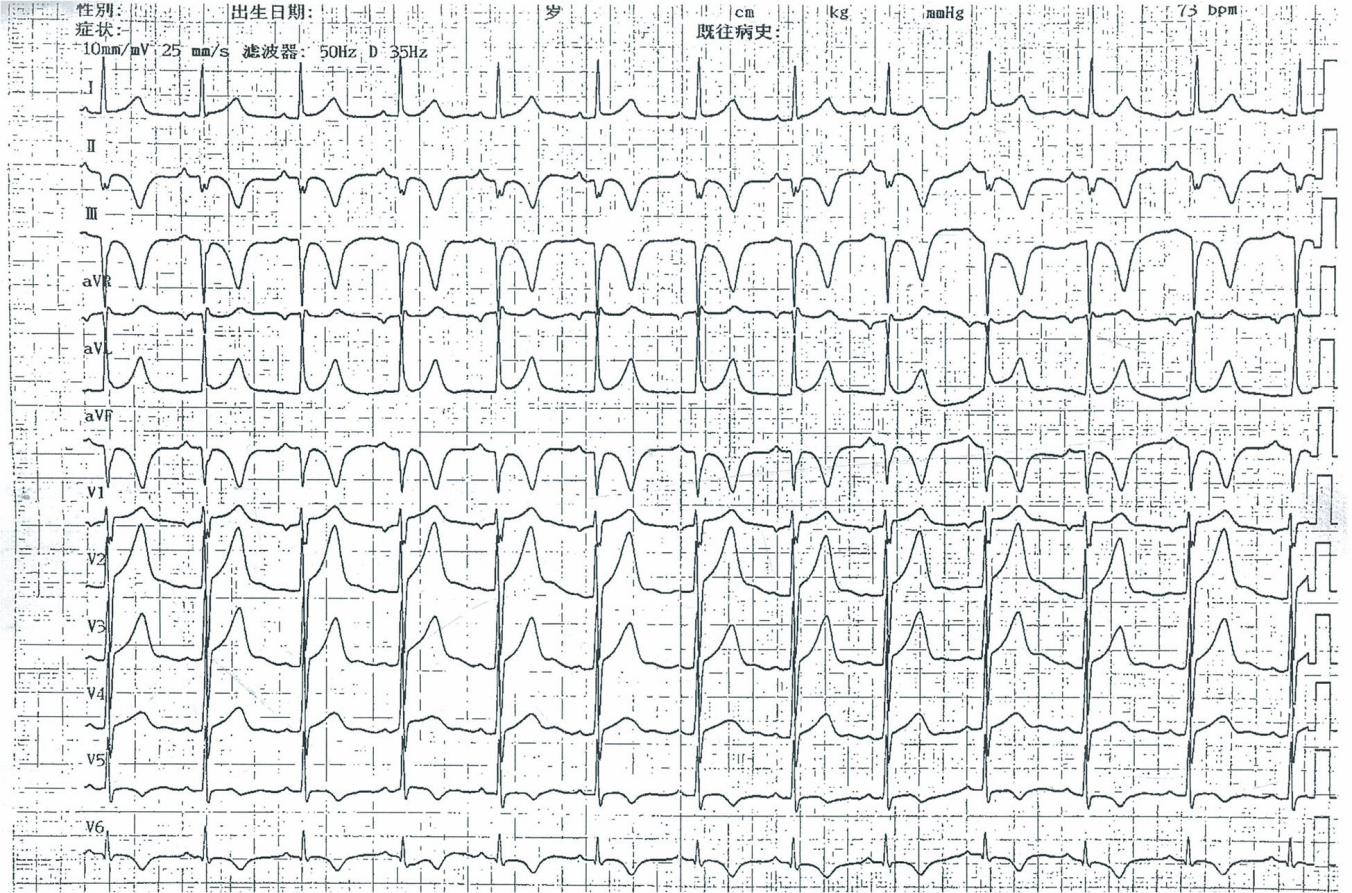
Electrocardiogram at discharge

**Fig. 4:**
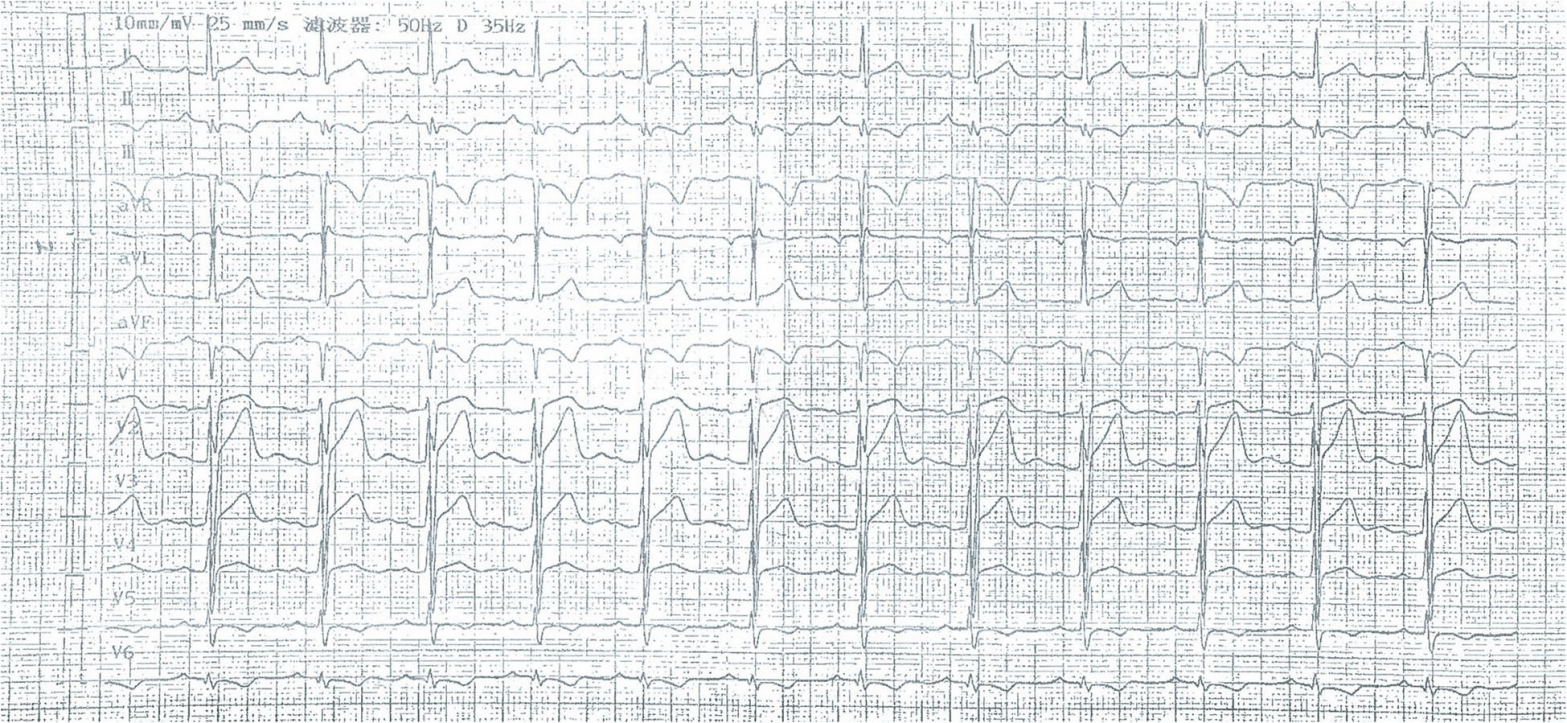
Electrocardiogram at 6-month follow-up

A 29-year-old male patient had presented with a history of 2-h chest pain and numbness of left upper arm before 5 days. The electrocardiogram (ECG) indicated acute inferior wall myocardial infarction (MI) and he refused any treatment at that time. Five days later he was admitted to our hospital for further examination. Physical examination showed no abnormal including arcus corneae and xanthelasma in eyelid, extensor tendon and achilles tendons. He had no histories of diabetes mellitus, hyperthyroidism, heart disease, hepatic or renal disease and no family history of FH. The ECG showed deep Q wave and inverted T wave in leads II, III and aVF (Fig. 1) and the echocardiogram revealed the diastolic dysfunction of left ventricular with a decreased LV ejection fraction (EF, 48%). The lower extremities ultrasound revealed atherosclerotic plaque in the posterior wall of right common femoral artery. Blood tests showed CK-MB of 21.4 U/L, lactate dehydrogenase of 452 U/L, hs-CRP of 71.2 ng/L, triglyceride (TG) (Triglyceride Kit method) of 0.88 mmol/L, total cholesterol (TC) of 6.87 mmol/L (Cholesterol Kitmethod), low density lipoprotein cholesterol (LDL-C) of 5.90 mmol/L and high density lipoprotein cholesterol (HDL-C) of 1.09 mmol/L (Direct Method-Surfactant Clearance Method).Further laboratory tests revealed highly elevated anticardiolipin antibody (ELISA method) of more than 120RU/ml (0-12RU/ml) and no other abnormal auto-antibodies, including β2-glicoprotein antibodies IgM, IgA, IgG, lupus anticoagulant (LA). DNA analysis for antiphospholipid antibody syndrome (APS) was not performed. Coronary artery angiography (CAG) demonstrated predominant right coronary artery (RCA) and diffuse lesions in the middle and distal segments of the left anterior descending (LAD) artery with the stenosis up to 40~50% (Fig. 2a) and total occlusive RCA from the middle segment (Fig. 2b) with LAD-RCA collateral circulation. With the treatments of anticoagulation (heparin), double antiplatelets (aspirin and ticagrelor) and lipid-modulating (rosuvastatin), he was implanted a stent at the middle segment of the RCA (Fig. 2c). Four days later, he was discharged without any complication. The ECG at discharge showed that the inverted T waves were deeper than those at admission in leads II, III and aVF (Fig. 3). At 6-month follow-up, the laboratory test showed the level of anticardiolipin antibody (ELISA method) was less than 2.0 RU/ml (0-12RU/ml). Lipid profile revealed TG of 0.98 mmol/L, TC of 6.22 mmol/L, LDL-C of 5.53 mmol/L and HDL-C of 0.99 mmol/L.CAG showed 70% in-stent restenosis of the RCA. ECG revealed deep Q waves and inverted T waves (Fig. 4). The patient looked good without any activity restriction and discomfort. At 8-month follow-up, the level of anticardiolipin antibody is less than 2.0 RU/ml and the lipid profile showed TG 0.64 mmol/L, TC 4.15 mmol/L, LDL-C 3.47 mmol/L and HDL-C 1.01 mmol/L.

## Discussion and conclusions

FH is a risk factor for premature ASCVD [16, 17] and antiphospholipid antibody is a contributor of thrombosis for myocardial infarction [14, 18]. According to the Dutch Lipid Clinic Network (DLCN) Diagnostic criteria (DLCN) [19], the patient was confirmed as possible FH with 5 scores including the premature coronary heart disease (2 points) and LDL-C level of 5.90 mmol/L (3 points). With the International Consensus Statement for Definite APS [20], we could not give a definite diagnosis of APS for this patient. However, the antiphospholipid antibodies can be present in the common population with bacterial or viral infection including HIV, HBV, HCV, herpes virus and parvovirus B19 [15, 21]. Infection-related antibodies will totally disappear once the infection is gone [22]. Although this patient did not have an obvious infectious manifestation before his admission, some occult infections could not be completely excluded. Therefore, transient elevation of anticardiolipin antibody could be a trigger or biomarker for MI in patients with FH [14, 18]; however, the causative effects of transient increased anticardiolipin antibody and MI still need further study.

### Limitations

We did not perform the genetic testing about the APS and the test of anticardiolipin antibody at 12-week follow-up.

## Data Availability

All the data in this study are included in the manuscript.

## Abbreviations

AMI: Acute myocardial infarction
aPL: Antiphospholipid antibody
APS: Antiphospholipid antibody syndrome
ASCVD: Atherosclerotic cardiovascular disease
CAD: Coronary artery disease
CK-MB: Creatine kinase isoenzyme
ECG: Electrocardiograph
FH: Familial hypercholesterolemia
HDL-C: High-density lipoprotein cholesterol
hs-CRP: High-sensitivity C-reactive protein
LDL-C: Low-density lipoprotein cholesterol
LDLR: LDL receptor
TC: Total cholesterol
TG: Triglyceride
